# Serum profile of transferrin isoforms in juvenile idiopathic arthritis: a preliminary study

**DOI:** 10.1007/s00296-018-4051-z

**Published:** 2018-05-14

**Authors:** Ewa Gruszewska, Magdalena Sienkiewicz, Paweł Abramowicz, Jerzy Konstantynowicz, Monika Gudowska-Sawczuk, Lech Chrostek, Bogdan Cylwik

**Affiliations:** 1 Department of Biochemical Diagnostics, Medical University of Bialystok, Waszyngtona 15A Street, 15-269 Białystok, Poland; 2 Department of Pediatric Laboratory Diagnostics, Medical University of Bialystok, Waszyngtona 17 Street, 15-274 Białystok, Poland; 3 Department of Pediatrics, Rheumatology, Immunology and Metabolic Bone Diseases, Medical University of Bialystok, Waszyngtona 17 Street, 15-274 Białystok, Poland

**Keywords:** Transferrin isoforms, Juvenile idiopathic arthritis, Capillary electrophoresis, Sialylation

## Abstract

It is reported that alterations in protein glycosylation are present in adult rheumatic diseases; however, the data related to pediatric rheumatic conditions are very scarce. The aim of this study was to assess the effect of juvenile idiopathic arthritis (JIA) on the serum glycosylation profile of transferrin isoforms. Twenty-five patients with different clinical forms of an active JIA and 22 healthy controls were studied. Serum samples were analyzed by capillary electrophoresis on MINICAP electrophoretic system (Sebia, France) to determine the levels of transferrin isoforms. In patients with JIA, tetrasialotransferrin (median 82.6%; range 68.8–99.5) concentration was lower (*P* = 0.032), and pentasialotransferrin (median 14%; range 0.5–31.2) was higher (*P* = 0.020) in comparison to controls (median 84.45; range 79.8–87.4; median 11.55; range 9.7–16.1, respectively). No significant correlations between concentration of transferrin isoforms and disease activity score (JADAS 27) or the degree of disability (VAS and CHAQ) were found. Erythrocyte sedimentation rate and CRP levels correlated positively with disialotransferrin (*R* = 0.493, *P* = 0.017; *R* = 0.850, *P* < 0.001, respectively) and pentasialotransferrin (*R* = 0.533, *P* = 0.006; *R* = 0.491, *P* = 0.045, respectively), and negatively with trisialotransferrin (*R* = − 0.546, *P* = 0.007; *R* = − 0.515, *P* = 0.049, respectively) and tetrasialotransferrin (*R* = − 0.436, *P* = 0.029; *R* = − 0.504, *P* = 0.039, respectively). This preliminary study shows the shifts in transferrin isoforms profile among patients with JIA. Our data indicate a potential clinical utility of the transferrin isoforms measurement, especially tetrasialotransferrin and pentasialotransferrin. Further prospective studies on larger groups of patients should be conducted to validate the results.

## Introduction

The occurrence of rheumatic diseases in children is much less common than in adults, although it is reported not infrequently [1, 2]. The prevalence rate found in different studies worldwide ranges from 30 to 100 per hundred thousand children [2, 3]. The most frequent chronic rheumatic condition in children and adolescents is juvenile idiopathic arthritis (JIA), being noninfectious inflammatory joint disease of unknown etiology, presenting with the onset before age of 16 years and with the minimum duration of symptoms exceeding 6 weeks [2, 4, 5]. According to the current criteria of International League Against Rheumatism (ILAR), the classification of JIA includes seven subtypes: oligoarthritis (oJIA), rheumatoid factor-negative polyarthritis (RF-pJIA), rheumatoid factor-positive polyarthritis (RF + pJIA), systemic-onset arthritis (sJIA), psoriatic arthritis (PsA), enthesitis-related arthritis (ERA) and undifferentiated arthritis (UA) [5, 6]. The pathogenesis of JIA is still poorly understood and, despite a great progress, the diagnosis is based primarily on clinical manifestations combined with ultrasonography, radiologic and laboratory abnormalities [5–7].

The majority of autoimmune rheumatic diseases, including JIA, are accompanied by acute or chronic inflammation process reflected by essential changes of serum concentration of acute phase proteins, which in fact are glycoproteins. The glycosylation profile of acute phase proteins in rheumatic diseases is variable and may demonstrate multiform changes. Alterations in the glycan structure of glycoproteins have been well documented in rheumatoid arthritis (RA) in adults [8, 9]. The most extensively studied and recognized disturbance associated with rheumatic diseases is an impaired glycosylation of immunoglobulin G (IgG) [10–12]. For example, observations of RA patients have shown increased amounts of agalactosyl isoforms of IgG, which strongly correlate with disease activity [10–12]. To identify glycosylation changes in JIA, a negative acute phase protein, i.e. transferrin was chosen. This multifunctional glycoprotein is composed of three structural subdomains: one polypeptide chain (containing 679 amino acids) and two carbohydrate chains [13], whereas each of these subdomains is characterized by a high variability (microheterogeneity). Nine isoforms of transferrin are currently known according to the number of sialic acid residues attached to the carbohydrate chains [13, 14]. Therefore, transferrin may specifically serve as a perfect research model for evaluation of the changes in glycosylation. The concentrations of transferrin and other acute phase proteins have been studied in a number of rheumatic diseases in both the adults and pediatric patients [9, 15]. However, the changes in the profile of transferrin isoforms were reported only in adult individuals with RA [16]. There are no published data on the alterations in transferrin isoforms in rheumatic diseases during growth. Therefore, the objective of this study was to evaluate the serum glycosylation profile of transferrin isoforms in juvenile idiopathic arthritis and to assess the potential diagnostic utility of the marker in JIA.

## Materials and methods

### Materials

#### JIA patients

Serum samples were obtained from 25 patients aged 9.3 ± 4.2 years with juvenile idiopathic arthritis (JIA) (7 males, 18 females) who were hospitalized in the Department of Pediatrics, Rheumatology, Immunology and Metabolic Bone Diseases (Bialystok University Children’s Clinical Hospital). All participants met the diagnostic ILAR criteria for JIA. The following clinical forms were determined among studied patients: 16 patients with oligoarthritis, 5 patients with RF-negative polyarthritis, 3 patients with systemic-onset polyarthritis course, and 1 patient with RF-positive polyarthritis. The disease duration ranged from 7 months to 14 years (median 3.5 years). There were no symptoms of glucocorticoid-induced adversity, and the clinical assessment of nutritional status remained normal. The Juvenile Arthritis Disease Activity Score (JADAS 27) was used for the assessment of the disease activity and symptom severity. The standard performance of JADAS 27 includes 4 domains: physician global assessment of disease activity, parent/patients global assessment of well-being, active joint count (cervical spine, elbows, wrists, metacarpophalangeal joints, proximal interphalangeal joints, hips, knees, and ankles), and erythrocyte sedimentation rate. A score range for this tool is from 0 (no disease activity) to 57. The functional status and the degree of disability were determined using Child Health Assessment Questionnaire (CHAQ; range 0–3; 0—best or no disability, 3—worst or severe disability) and a 10-cm Visual Analogue Scale (VAS) in which 0 denominates no activity/no pain, while the value of 10 indicates maximum activity or extremely severe pain. All participants were screened using standard methods for liver function, renal diseases and hematological disorders. No abnormalities were found in aminotransferases activity, GGTP, fasting glycaemia, creatinine level and GFR in either of studied groups. The hematological parameters, including hematocrit, hemoglobin and erythrocyte count were within the normal range in all children included in the study.

#### Control group

The control group was composed of serum samples collected from 22 age-matched healthy subjects who agreed to participate (10 males, 12 females; mean age 10.3 ± 4.3 years) without any symptoms of connective tissue disease, as ascertained by certified rheumatologist staff. The controls were derived from the outpatient clinic where they were followed for irrelevant minor health issues. The control subjects were screened for inflammatory disease and/or infection at the time of examination and were enrolled based on normal results of C-reactive protein level, erythrocyte sedimentation rate and complete blood count. None of the controls had hepatic impairment, renal disease, anemia or other hematological abnormalities. Written informed consent was obtained from all participants and their parents or legal guardians. The study was carried out in accordance with Helsinki Declaration and was approved by the Bioethical Committee at Medical University of Bialystok (Approval no. R-I-002/72/2016; 31.03.2016).

#### Sample collection

Blood samples from patients with juvenile idiopathic arthritis and controls were collected by peripheral vein puncture. The sera were separated immediately by centrifugation at 1500×*g* from 10 min at room temperature and stored at − 86 °C till the time of analysis.

### Methods

#### Determination of transferrin isoforms

The analysis of transferrin isoforms was performed on a MINICAP electrophoretic system using the MINICAP CDT reagent kit (Sebia; Evry, France). The MINICAP system uses the capillary electrophoresis (CE) in free solution. The system performs all sequences automatically to obtain transferrin isoform profile for quantitative analysis of each fraction. The human serum transferrin isoforms were separated into five major fractions according to their sialylation level: asialotransferrin, disialotransferrin, trisialotransferrin, tetrasialotransferrin, and pentasialotransferrin.

#### Statistical analysis

To check the normality of distribution, the Shapiro–Wilk test was used. Only trisialotransferrin showed normal distribution (*W* = 0.976, *P* = 0.827), the remaining isoforms: disialo-, tetrasialo- and pentasialotransferrin presented non-normal distribution (*P* < 0.001 for all). Because the majority of variables were distributed not normally, non-parametric tests were applied in the analyses. Results were expressed as median and range. The differences between studied and control groups were evaluated with Mann–Whitney *U* test. The correlations between transferrin isoforms, other laboratory tests, and disease activity scores were analyzed using Spearman’s correlation. The *P* value < 0.05 was considered as statistically significant.

## Results

The characteristics of patients with JIA and controls are presented in Table 1. In patients with JIA, the results of all tests were found to be significantly different from those in controls, except RF, MCV, MCH, and WBC.

**Table 1:** The characteristics of patients with juvenile idiopathic arthritis and control group

	JIA *N* = 25	Control group *N* = 22	*P*
ESR (mm/h)	23 2–95	3 2–17	< 0.001
CRP (mg/L)	3.99 0.13–71	0.53 0.06–5.7	0.005
RF (IU/mL)	10 6.7–92.9	9.55 7.4–12.2	0.920
RBC (10^6^/µL)	4.4 3.86–5.64	4.9 4.14–6.2	0.007
HGB (g/dL)	11.7 9.5–14.4	13.3 11.3–17.7	< 0.001
HCT (%)	35.6 31.2–43.2	39.25 34.2–50.6	0.002
MCV (fL)	78.6 61.7–89.1	79.65 73.5–88.9	0.977
MCH (pg)	26.1 18.8–30.0	27.35 24.8–29.3	0.106
MCHC (g/dL)	33.3 30.4–34.5	34.25 31.6–35.9	0.001
WBC (10^3^/µL)	8.06 3.45–23.72	6.55 4.47–11.93	0.100
PLT (10^3^/µL)	351 178–1033	279.5 126–475	0.009
JADAS 27	9.40 0.2–51.0	–	
CHAQ	0.89 0.02–2.69	–	
VAS	3.60 0.2–10	–	

Data are median and ranges. The differences between studied patients and controls were estimated by Mann–Whitney *U* test

*JIA* juvenile idiopathic arthritis, *ESR* erythrocyte sedimentation rate, *CRP* C-reactive protein, *RF* rheumatoid factor, *RBC* red blood cells, *HGB* hemoglobin, *HCT* hematocrit, *MCV* mean corpuscular volume, *MCH* mean corpuscular hemoglobin, *MCHC* mean corpuscular concentration, *WBC* white blood cells, *PLT* platelets, *JADAS 27* Juvenile Arthritis Disease Activity Score in 27 joints, *CHAQ* Childhood Health Assessment Questionnaire, *VAS* Visual Analogue Scale

The serum concentrations of transferrin isoforms in patients with JIA and healthy subjects were adjusted and expressed as percentage (Table 2). Differences in the relative concentration of tetrasialotransferrin and pentasialotransferrin were found between patients with JIA and healthy subjects. Mean concentration of tetrasialotransferrin was significantly lower (*Z* = − 2.143, *P* = 0.032), and of pentasialotransferrin was significantly higher compared with controls (*Z* = 2.325, *P* = 0.020).

**Table 2:** The serum concentration of transferrin isoforms in patients with juvenile idiopathic arthritis and controls

	Disialotransferrin (%)	Trisialotransferrin (%)	Tetrasialotransferrin (%)	Pentasialotransferrin (%)
Control group *N* = 22	0.6 0.3–1.0	2.85 0.9–7.2	84.45 79.8–87.4	11.55 9.7–16.1
JIA *N* = 25	0.7 0.4–2.1	2.5 0.6–4.8	82.6 68.8–99.5	14.0 0.5–31.2
	*P* = 0.146	*P* = 0.346	*P* = 0.032*	*P* = 0.020*

Data are median and ranges. The differences between studied patients and controls were estimated by Mann–Whitney *U* test

*JIA* juvenile idiopathic arthritis

*Significant differences in comparison with the control group

In patients with JIA, no significant correlation between transferrin isoforms and disease activity score JADAS 27 was found, and no such correlation was observed regarding the degree of disability assessed by standard tools CHAQ and VAS either (*P* > 0.05 for all comparisons) (Table 3).

**Table 3:** Spearman’s correlation between the transferrin isoforms and the disease activity scores, the degree of disability scores and laboratory test results

	Disialotransferrin (%)	Trisialotransferrin (%)	Tetrasialotransferrin (%)	Pentasialotranferrin (%)
ESR (mm/h)	*R* = 0.493 *P* = 0.017	*R* = − 0.546 *P* = 0.007	*R* = − 0.436 *P* = 0.029	*R* = 0.533 *P* = 0.006
CRP (mg/L)	*R* = 0.850 *P* < 0.001	*R* = − 0.515 *P* = 0.049	*R* = − 0.504 *P* = 0.039	*R* = 0.491 *P* = 0.045
RF (IU/mL)	*R* = 0.383 *P* = 0.309	*R* = − 0.517 *P* = 0.154	*R* = 0.009 *P* = 0.979	*R* = 0.158 *P* = 0.639
RBC (10^6^/µL)	*R* = 0.621 *P* = 0.013	*R* = − 0.154 *P* = 0.584	*R* = − 0.390 *P* = 0.121	*R* = 0.439 *P* = 0.078
HGB (g/dL)	*R* = − 0.001 *P* = 0.997	*R* = 0.291 *P* = 0.292	*R* = 0.162 *P* = 0.534	*R* = − 0.136 *P* = 0.602
HCT (%)	*R* = 0.028 *P* = 0.920	*R* = 0.328 *P* = 0.233	*R* = 0.037 *P* = 0.888	*R* = − 0.038 *P* = 0.885
MCV (fL)	*R* = − 0.418 *P* = 0.121	*R* = 0.302 *P* = 0.273	*R* = 0.412 *P* = 0.100	*R* = − 0.458 *P* = 0.065
MCH (pg)	*R* = − 0.440 *P* = 0.100	*R* = 0.242 *P* = 0.385	*R* = 0.489 *P* = 0.046	*R* = − 0.497 *P* = 0.042
MCHC (g/dL)	*R* = − 0.329 *P* = 0.231	*R* = 0.119 *P* = 0.672	*R* = 0.583 *P* = 0.014	*R* = − 0.503 *P* = 0.039
WBC (10^3^/µL)	*R* = 0.624 *P* = 0.013	*R* = − 0.451 *P* = 0.092	*R* = − 0.463 *P* = 0.061	*R* = 0.482 *P* = 0.050
PLT (10^3^/µL)	*R* = 0.807 *P* < 0.001	*R* = − 0.581 *P* = 0.023	*R* = − 0.402 *P* = 0.110	*R* = 0.487 *P* = 0.047
JADAS 27	*R* = 0.239 *P* = 0.272	*R* = − 0.355 *P* = 0.097	*R* = − 0.301 *P* = 0.143	*R* = 0.318 *P* = 0.121
CHAQ	*R* = 0.140 *P* = 0.524	*R* = − 0.323 *P* = 0.132	*R* = − 0.263 *P* = 0.204	*R* = 0.300 *P* = 0.144
VAS	*R* = 0.104 *P* = 0.637	*R* = − 0.343 *P* = 0.109	*R* = − 0.159 *P* = 0.448	*R* = 0.207 *P* = 0.322

*ESR* erythrocyte sedimentation rate, *CRP* C-reactive protein, *RF* rheumatoid factor, *RBC* red blood cells, *HGB* hemoglobin, *HCT* hematocrit, *MCV* mean corpuscular volume, *MCH* mean corpuscular hemoglobin, *MCHC* mean corpuscular concentration, *WBC* white blood cells, *PLT* platelets, *JADAS 27* Juvenile Arthritis Disease Activity Score in 27 joints, *CHAQ* Childhood Health Assessment Questionnaire, *VAS* Visual Analogue Scale

In patients with JIA, the level of ESR and CRP correlated significantly with all transferrin isoforms (Table 3). There were positive correlations with disialotransferrin and with pentasialotransferrin, whereas negative correlations with trisialotransferrin and with tetrasialotransferrin were observed. There were also positive correlations for disialotransferrin and pentasialotransferrin with PLT, and with WBC. Moreover, there were positive correlations for MCH and MCHC with tetrasialotransferrin, and negative correlations with pentasialotransferrin.

## Discussion

In the present study, the glycosylation profile of transferrin isoforms was determined in a spectrum of juvenile idiopathic arthritis, using the capillary electrophoresis technology. This method has been already ascertained as highly consistent with the HPLC, recognized and widely accepted as a reference method. To comply with a uniform and convenient manner of data presentation, the results were expressed by percentage of total transferrin concentration as reported elsewhere [17].

This study provides evidence of the significant changes in glycosylation profile of transferrin in patients with JIA. The major alterations were observed in relation to tetrasialotransferrin and pentasialotransferrin isoforms. In healthy individuals, the tetrasialotransferrin is the greatest fraction of transferrin and reaches the level of 64–80%, while pentasialotransferrin usually reaches the level of 15% [18, 19]. In our study, the JIA children and adolescents demonstrated a significant decrease in tetrasialotransferrin when compared with controls. In contrast, pentasialotransferrin was significantly higher. Beyond some relevant studies on the IgG, there is a scarce number of published reports about alterations in the glycosylation of glycoproteins in rheumatic diseases, particularly in children [9–11]. This study, although of cross-sectional and preliminary nature, is the first one concerning the specific topic. Importantly, the increase in pentasialotransferrin is in accordance with available literature data. The increase of high branching isoforms may be explained by aberrant activity of enzymes involved in branching of glycans, e.g., an elevation in activity of *N*-acetylglucosaminyltransferase V (GnT-V), which is responsible for the β1–6 branching to N-glycans [12, 20]. This has been reported in a study by Feelders et al., who showed an increase in transferrin branching in chronic inflammatory diseases, including rheumatoid arthritis [16]. The increased branching of glycans ending with sialic acid is often associated with an increased sialylation in many other diseases. This supports our observations in such a way as an explicitly increased concentration of sialic acid was also previously reported in an adult population with rheumatic diseases [21].

In the present study, no correlation was observed between the disease activity assessed using JADAS 27 or the degree of disability ascertained by CHAQ and VAS either. This may have been somewhat related to a limited number of participants, and the above methodological issue led to a difficulty in patient stratification into specific groups assigned to clinical subtypes of JIA. Such an association may presumably be dependent on the subtypes of JIA or disease severity. Although the observation was inconclusive, there is a possibility that further analyses on larger populations would allow elucidating the effects of individual subtypes of JIA. Furthermore, our data showed that the classic markers of inflammation, i.e. ESR and CRP were associated with pentasialotransferrin in children with JIA, whereas correlated inversely with tetrasialotransferrin concentration. It is acknowledged that the erythrocyte sedimentation rate and C-reactive protein levels are routinely used as standard laboratory measures of disease activity, alongside the general indicators of inflammation including elevated levels of WBC and PLT, and decreased RBC. We demonstrated a quantitative association between a high pentasialotransferrin concentration and both the greater leukocytosis and higher platelet count. These findings suggest that the tetrasialotransferrin and pentasialotransferrin isoforms may be helpful in assessing an inflammatory activity of the disease, even if the routine examination seems yet to be unfeasible. Interestingly, the transferrin isoforms, especially tetrasialotransferrin and pentasialotransferrin, appear to be more sensitive and more useful markers than the ESR and CRP for inflammatory activity. They reflect changes at the molecular level, before any apparent increase in the ESR and CRP concentration in the blood occurs. Nevertheless, well-designed prospective research should be conducted in the future for verification of these pilot findings, and to address the potential role of transferrin isoforms in disease control and long-term monitoring. Noticeably, several ongoing studies demonstrate an approach to identify new diagnostic targets in JIA. Some novel biomarkers of the disease and inflammation have been extensively studied showing potential clinical utility in early stages of JIA, although the results remain inconsistent [22–24]. As this is a challenging issue in pediatric rheumatology, the evaluation of transferrin isoforms may also contribute to extend the diagnostic tools, given that results of the present study are promising.

To summarize, this pilot study shows a specific profile of transferrin isoforms in patients with juvenile idiopathic arthritis. These results, though based on a small sample size, indicate the potential diagnostic utility of the measurement of transferrin isoforms in JIA, especially tetrasialotransferrin and pentasialotransferrin. However, further prospective cohort studies are needed to elucidate reliability of these findings for clinical practice.
